# A referable clinical pattern of spondyloarthritis-associated uveitis

**Published:** 2018

**Authors:** Costin Traian Mitulescu, Claudiu Costinel Popescu, Constantin Laurențiu Oprea, Denisa Predețeanu, Sînziana Istrate, Radu Ciuluvică, Liliana-Mary Voinea

**Affiliations:** * Ophthalmology Department, University Emergency Hospital, Bucharest, Romania; ** “Carol Davila” University of Medicine and Pharmacy, Bucharest, Romania; *** “Dr. Ion Stoia” Clinical Centre for Rheumatic Diseases, Bucharest, Romania; **** Ophthalmology Department, “Sfântul Apostol Andrei” Clinical County Emergency Hospital, Constanța, Romania; ***** “Sfânta Maria” Clinical Hospital, Bucharest, Romania

**Keywords:** uveitis, spondyloarthritis, ankylosing spondylitis

## Abstract

**Objective.** The aim of the study was to identify a clinical pattern of spondyloarthritis (SpA)-associated uveitis in order to facilitate proper rheumatologic referral by ophthalmologists.

**Methods.** Demographic data were recorded and acute phase reactants were measured using standard methods between May and August 2017, for all adult patients (age > 18 years) presenting with acute uveitis (standardization of uveitis nomenclature). Afterwards, they were referred to a rheumatology clinic to be screened for the 2009 Assessment of SpondyloArthritis International Society (ASAS) classification criteria of SpA. SpA and non-SpA patients were then compared using appropriate statistical tests (significant if p < 0.05). All the patients offered a written informed consent and the study was approved by the local ethics committee.

**Results.** The sample included 67 subjects, of whom 32 (47.8%) were classified with SpA, of whom 21 were known with this diagnosis (median disease duration of 13.6 years), while 11 (34.4% of the SpA subgroup) fulfilled the ASAS classification criteria for SpA without a prior diagnosis. Compared to non-SpA patients, SpA patients were more frequently male urban dwellers, they were significantly younger, they had a higher prevalence of HLA-B27 and recurrent uveitis, and they had higher median of C-reactive protein and lower median of white blood counts.

**Conclusions.** Uveitis is an important revelatory manifestation of SpA. Young men presenting in an ophthalmology department with acute anterior uveitis and inflammatory back pain should be referred to a rheumatology unit.

**Abbreviations:** AAU = Acute Anterior Uveitis, AS = Ankylosing Spondylitis, ASAS = Assessment Of Spondyloarthritis International Society, CRP = C-Reactive Protein, ESR = Erythrocyte Sedimentation Rate, HLA = Human Leukocyte Antigen, IBP = Inflammatory Back Pain, IBD = Inflammatory Bowel Disease, IL = Interleukin, MRI = Magnetic Resonance Imaging, Nsaids = Non-Steroidal Anti-Inflammatory Drugs, SD = Standard Deviation, Spa = Spondyloarthritis, Tnfα = Tumour Necrosis Factor Alpha, WBC = White Blood Count.

## Introduction

Spondyloarthritis (SpA) is a group of chronic inflammatory diseases which have common genetic predisposition (HLA-B27) and clinical features such as chronic inflammatory back pain (IBP) revealed by sacroiliac bone oedema and sacroiliitis in young adults, sometimes with asymmetric oligo-articular involvement of large joints. The SpA group typically includes ankylosing spondylitis (AS), psoriatic arthritis, reactive arthritis, inflammatory bowel disease (IBD)-associated SpA and undifferentiated spondyloarthritis [**1**].

The Assessment of Spondyloarthritis International Society (ASAS) proposed classification criteria for axial SpA patients [**1**,**2**]: patients aged less than 45 years with back pain for more than 3 months can be categorized with axial SpA if they have either sacroiliitis on imaging and at least one SpA feature, either HLA-B27 or at least two other SpA features. Sacroiliitis is defined either as bilateral grade 2–4 or as unilateral grade 3–4 sacroiliitis on standard radiographs, fulfilling the modified New York criteria [**3**], either sacroiliac bone marrow oedema/ osteitis on magnetic resonance imaging (MRI). The cited SpA features include inflammatory back pain, arthritis, uveitis, heel enthesitis, dactylitis, psoriasis, inflammatory bowel disease, good response to non-steroidal anti-inflammatory drugs (NSAIDs), and family history of SpA, HLA-B27, and elevated C - reactive protein (CRP). The group of axial SpA includes non-radiographic axial SpA with bone oedema revealed by MRI and AS with sacroiliitis evident on standard radiographs. ASAS also proposed classification criteria for peripheral SpA [**4**]: a patient with arthritis or enthesitis or dactylitis is classified with peripheral SpA if he or she has at least one of the following criteria: psoriasis, IBD, preceding infection, HLA-B27, uveitis, sacroiliitis on imaging (radiographs or MRI); or at least two of the following criteria: arthritis, enthesitis, dactylitis, IBP in the past, positive family history for SpA. The diagnosis of peripheral SpA is used until the patient fulfils the classification criteria for a definite disease from the SpA group.

The most frequent extra-articular manifestation of SpA is recurrent unilateral acute anterior uveitis (AAU), present in up to 40-50% of the patients during the disease course and associated with the presence of HLA-B27 and the general absence of long-term sequelae [**5**,**6**]. The genetic predisposition of uveitis in SpA reaches beyond HLA-B27, since genome-wide scans have identified other significant predisposing loci (e.g. IL23R, ERAP1, chromosome 9p21) [**7**,**8**]. However, there are many other causes of uveitis, including infections (e.g. toxoplasmosis, herpes, varicella-zoster, tuberculosis, human immunodeficiency virus, syphilis etc.), trauma (injury, post-surgical), neoplasia (e.g. lymphoma, leukemia), medications (e.g. quinolones, vaccines, etc.), immune-mediated diseases (e.g. HLA-B27-associated uveitis, Behçet’s disease, systemic lupus erythematosus, sarcoidosis, Lyme disease, etc.).

Since uveitis has a very strong effect of the quality of life [**9**], the affected patients present themselves to the ophthalmologist, even those with unattended chronic back pain self-treated with over-the counter NSAIDs. When confronted with a case of acute uveitis, the ophthalmologist should initiate the process of differential diagnosis. If an immune-mediated mechanism is suspected, a rheumatology consult is needed knowing that, on one hand, SpA is the most frequent immune-mediated disease causing uveitis and, on the other hand, uveitis is a significant risk factor for SpA development [**10**,**11**], especially if it is recurrent [**12**]. If indeed the uveitis episode proves to be an early or a revealing extra-articular manifestation of SpA, then the prompt management of this potentially debilitating disease can prevent loss of musculoskeletal function. Moreover, since the association of HLA-B27 and AS is proved to entail higher risks of complications in uveitis patients [**13**], the ophthalmologist should adapt the management scheme in order to prevent possible visual disability.

Observational studies have underlined the essential role of ophthalmologists in diagnosing underlying SpA (the study of one cohort reports that more the half of uveitis patients fulfilled SpA classification criteria [**14**]). In this context, the study aims to identify a clinical pattern of SpA-associated uveitis in order to facilitate a proper rheumatologic referral by ophthalmologists.

## Methods

**Patients**

The study was designed to record clinical data in a cross-sectional model. All the patients who presented in the random order of healthcare access to the Ophthalmology Department of University Emergency Hospital in Bucharest between May and August 2017 were screened for inclusion in the study. The inclusion criterion was a diagnosis of uveitis made by each attending ophthalmologist on the screening visit. Patients under the age of 18 years and patients with chronic forms of uveitis were excluded from the study. After a clinical evaluation (diagnosis and treatment of uveitis), the included patients were referred to the Rheumatology Department of “Sfânta Maria” Clinical Hospital (Bucharest) within two working days to either confirm a diagnosis of SpA from the patient’s history or screen for undiagnosed SpA. Each patient offered a written informed consent for the use of clinical data on the hospital visits (ophthalmology and rheumatology) and the study was approved by the local ethics committee.

**Variables**

Upon presentation to the Ophthalmology Department, demographic data were recorded (age, gender, dwelling) and the patients underwent routine laboratory measurements of four acute phase reactants: erythrocyte sedimentation rate (ESR; normal < 20 mm/ h, Westergren method), serum fibrinogen (normal < 490 mg/ dL, enzyme-linked immunoabsorbant assay), CRP (normal < 5 mg/ L, nephelometry) and white blood count (WBC; normal < 104/ μL, automated counter). In order to be included in the study, the diagnosed uveitis must have fulfilled the standardization of uveitis nomenclature: limited (≤ 3 months duration); acute (sudden onset and limited duration); not chronic (persistent uveitis with relapse in less than 3 months after discontinuing treatment); anterior uveitis that affects the anterior chamber (iritis, iridocyclitis, anterior cyclitis); intermediate uveitis that affects the vitreous (pars planitis, posterior cyclitis, hyalitis); posterior uveitis that affects retina or choroid (focal, multifocal, or diffuse choroiditis; chorioretinitis; retinochoroiditis; retinitis; neuroretinitis); panuveitis that affects the anterior chamber, the vitreous and the retina or choroid [**15**]. Recurrent episodes of uveitis were defined according to the same nomenclature: repeated episodes separated by periods of inactivity without treatment for more than 3 months. Patients were classified with SpA if they fulfilled either the 2009 ASAS criteria [**1**,**2**]: back pain for more than 3 months with onset before 45 years of age with either sacroiliitis on imaging (resonance magnetic imaging or conventional radiography) and at least 1 SpA feature (IBP, arthritis, heel enthesitis, uveitis, dactylitis, psoriasis, inflammatory bowel disease, good response to NSAIDs, family history of SpA, positive HLA-B27, elevated CRP) or positive HLA-B27 and at least two other SpA features.

**Statistics**

Normally distributed continuous variables were reported as “mean ± standard deviation”, non-normally distributed continuous variables were reported as “median (minimum-maximum)”, while qualitative variables were expressed as “absolute frequency (proportion of group)”. Distribution normality was assessed using descriptive statistics, normality plots and the Kolmogorov-Smirnov tests.

The comparison of continuous variables (e.g. ESR) among the subgroups (controls, SpA) was assessed using the Mann-Whitney U tests. The prevalence of nominal variables (e.g. HLA B27) among subgroups (controls, SpA) was evaluated using the χ2 tests. All tests were considered significant if their calculated p values were below 0.05 and were done using IBM SPSS v.20 (IBM Inc., Armonk, New York, 2010) for Windows.

## Results

**General characteristics**

The sample included 67 subjects, of which 32 (47.8%) were classified with SpA (**Table 1**). Of the 32 cases of confirmed SpA, 21 patients were known with this diagnosis (or a diagnosis of AS) from their medical history, having a median disease duration of 13.6 (2-38) years, while 11 patients (16.7% of the total sample; 34.4% of the SpA subgroup) fulfilled the ASAS classification criteria for SpA without a prior diagnosis.

**Table 1:** General characteristics of the sample and subgroup comparison

	*all*	*control*	*SpA*	**
	*(n = 67)*	*(n = 35)*	*(n = 32)*	*p*
age (years)	48.1 ± 16.7	53 (18-83)	38 (26-72)	0.034*
male gender (n, %)	41 (60.3%)	11 (31.4%)	30 (93.8%)	< 0.001#
urban dwelling (n, %)	43 (64.2%)	20 (57.1%)	23 (71.9%)	0.039#
HLA-B27 + (n, %)	38 (56.7%)	7 (20.0%)	31 (96.9%)	< 0.001#
AAU (n, %)	52 (77.6%)	25 (71.4%)	27 (84.4%)	0.046#
recurrent (n, %)	30 (44.8%)	7 (20.0%)	23 (71.9%)	< 0.001#
ESR (mm/h)	16 (2-109)	17 (4-59)	16 (2-109)	0.857*
fibrinogen (mg/dL)	400 (200-789)	393 (235-528)	410 (200-789)	0.568*
CRP (mg/L)	2.75 (0.1-142)	0.6 (0.1-22.1)	7.6 (0.4-142.0)	< 0.001*
WBC (x 103/μL)	8.3 (4.0-22.0)	9.4 (4.4-16.1)	8.0 (4.0-13.0)	0.035*
*Notes:* “age” of the entire sample was distributed normally and it is reported as “mean ± SD”; “age” in subgroups and all inflammatory markers (ESR, fibrinogen, CRP, WBC) were distributed non-normally and they are reported as “median (minimum – maximum)”; nominal variables are reported as “absolute frequency (proportion of group)”; p values represent the significance of Mann-Whitney U tests (*) or χ2 tests (#).				
*Abbreviations:* AAU = acute anterior uveitis; CRP = C-reactive protein; ESR = erythrocyte sedimentation rate; HLA = human leukocyte antigen; SD = standard deviation; SpA = spondyloarthritis; WBC = white blood count; + = positive/present.				

Differences between controls and SpA patients

Compared to non-SpA patients (**Table 1**), those classified with SpA were more frequently male urban dwellers, significantly younger, having a higher prevalence of HLA-B27 and recurrent AAU. 

**Fig. 1:**
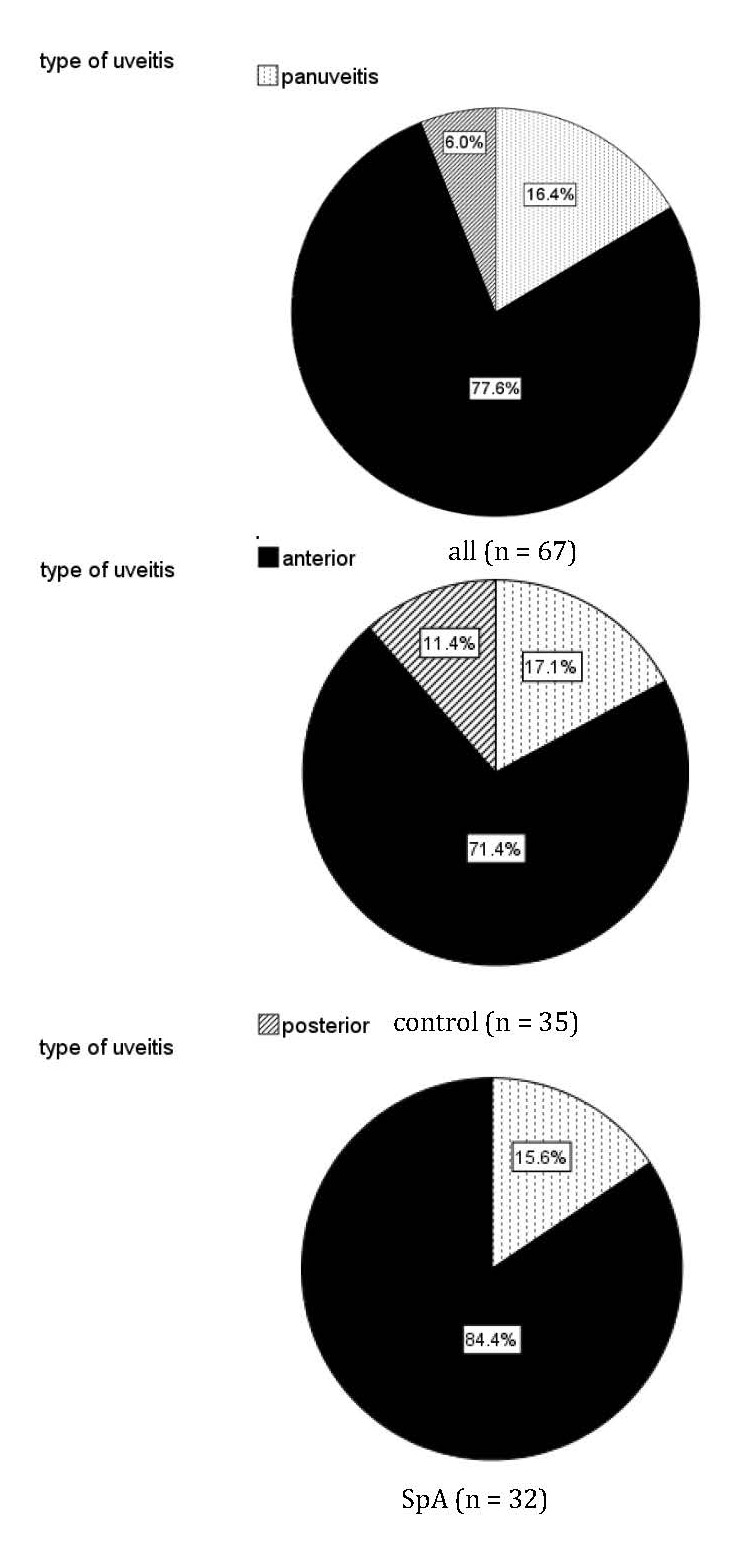
The distribution of uveitis type (anterior, posterior, panuveitis) in the sample and its subgroups (controls and spondyloarthritis - SpA)

**Fig. 2:**
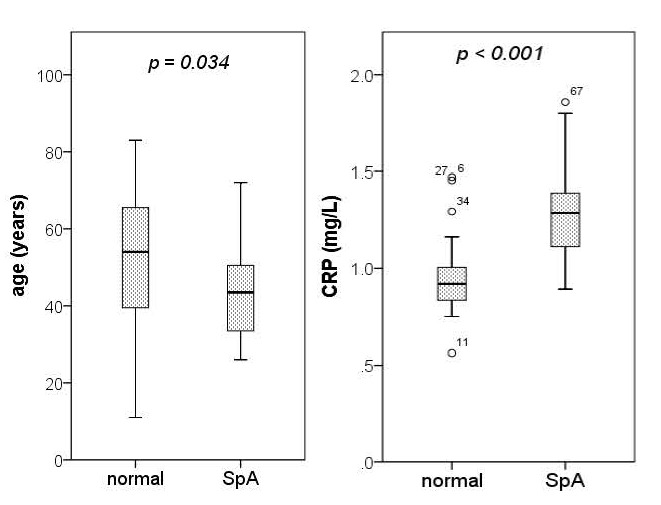
The differences of age and C-reactive protein (CRP) between controls (n = 35) and spondyloarthritis (SpA) patients (n = 32; p values represent the significance of Mann-Whitney U tests)

What is important to note is that there were no cases of intermediate uveitis in the whole sample and there were no cases of posterior uveitis in the SpA subgroup (**Fig. 1**). Regarding inflammatory markers, compared to normal patients, those classified with SpA had a significantly higher median of CRP (**Fig. 2**) and a significantly lower median of WBC, with no significant differences regarding ESR and serum fibrinogen. 

## Discussion

The study aimed to identify a clinical pattern of SpA-associated uveitis in order to facilitate proper rheumatologic referral by ophthalmologists. In this sense, recurrent AAU in men under 40 years (as previously reported [**16**]), with very high CRP and normal WBC seems to characterize SpA-associated uveitis. The association of recurrent uveitis with male gender and young age was previously reported in literature [**17**], but to our knowledge, the association with higher CRP and normal WBC was not previously reported. Since locally infiltrated inflammatory cells in the uveal tract do not usually organize themselves as lymphoid nodes and since there are no local cells with inducible or pre-designed capacity to produce pro-inflammatory cytokines (like synovial cells in peripheral joints), we hypothesize that the association of uveitis with higher CRP levels is not an expression of the local eye involvement but merely a part of a systemic involvement, which includes active IBP and possibly peripheral arthritis. This hypothesis is supported by another novel association we reported, namely the low/ normal WBC in our patients. If this is true, eye involvement in SpA seems be a form of collateral immunological damage of a differently targeted pathogenic process. As Rosenbaum and Rosenzweig observed in 2012 [**18**], uveitis and spondylitis are antagonists: uveitis has an acute onset, spondylitis onsets insidiously; uveitis is usually unilateral, spondylitis is usually bilateral; uveitis usually heals between attacks, spondylitis never resolves. We would describe the relationship of uveitis to SpA as that of gout to the metabolic syndrome [**19**]. As SpA encompasses a wide phenotype range of clinical manifestations (e.g. arthritis, uveitis, enthesitis, dactylitis, psoriasis, IBD) which occur both in relationship with HLA-B27 and outside of it, we can assume that the responsible cause is a yet an unidentified immune response defect that manifests itself differently according to the presence or absence of local and systemic risk factors.

There have been systematic attempts to standardize the referral of uveitis patients by ophthalmologists to rheumatologists. For example, the DUET (Dublin Uveitis Evaluation Tool) algorithm proposed that HLA-B27 should be tested in patients with AAU and back pain (age of onset under 45 years with a duration more than 3 months) or joint paint requiring a general-practitioner visit; if HLA-B27 is present or if the patient has current or past psoriasis, then he or she should be referred to the rheumatologist [**20**,**21**]. Our results showed that 20% of non-SpA patients and almost all of the SpA patients with uveitis had the HLA-B27 gene (**Table 1**). 

In these circumstances, we advocate the referral of AAU patients with IBP without HLA-B27 testing (which could be tested as recommended by the rheumatologist). According to the Berlin criteria, IBP is defined by at least 2 of the following 4 criteria: morning stiffness of more than 30 minutes; improvement with exercise but not with rest; awakening because of back pain during the second half of the night only; alternating buttock pain [**22**]. More recently, ASAS has defined IBP if 4 of the following 5 criteria are met: age at onset below 40 years; insidious onset; improvement with exercise; no improvement with rest; nocturnal back pain [**23**]. These questions can be easily accommodated to fit the clinical interview by an ophthalmologist with the occasion of a consult for AAU and the patient should be referred to a rheumatologist, since the presence of IBP alone is sufficient reason for SpA differential diagnosis (a recent study showed that there is a 30% probability of SpA at 10 years for patients with IBP [**24**]). This approach is supported by encouraging observational research: Wach et al. reported that the Berlin criteria had 61.9% sensitivity and 97.5% specificity in diagnosing SpA in patients presenting with uveitis and back pain [**25**]. Also, it is a fact that the most frequent form in SpA, in which uveitis appears, is AS, and that uveitis may precede this diagnosis, may be concomitant with this diagnosis or it may appear anytime during the disease course [**26**] (the cumulative incidence of new uveitis over 2 years in a SpA cohort was 3.1% [**27**]), even as a paradoxical manifestation of tumour necrosis factor alpha (TNFα) inhibitors [**28**].

In conclusion, uveitis is an important revelatory manifestation of SpA. Ophthalmologists should know the definition of IBP, since young men presenting in an ophthalmology department with AAU and IBP should be referred to a rheumatology unit. 

**Conflict of interests**

The authors declare there is no conflict of interest. The authors have equal contribution in this study.

## References

[1] Mitulescu TC, Voinea LM, Predeteanu D, Banica LM, Stavaru C, Matache C (2014). Abnormalities in soluble CD147/MMPs/TIMPs axis in Ankylosing Spondylitis patients with and without a history of Acute Anterior Uveitis/ Anomalii ale axei CD147 solubil/MMPs/TIMPs la pacienții cu spondilită anchilozantă cu sau fără uveită acută anterioară. Romanian Review of Laboratory Medicine.

[2] Rudwaleit M, Landewe R, van der Heijde D, Listing J, Brandt J, Braun J, Burgos-Vargas R, Collantes-Estevez E, Davis J, Dijkmans B (2009). The development of Assessment of SpondyloArthritis international Society classification criteria for axial spondyloarthritis (part I): classification of paper patients by expert opinion including uncertainty appraisal. Ann Rheum Dis.

[3] Rudwaleit M, van der Heijde D, Landewe R, Listing J, Akkoc N, Brandt J, Braun J, Chou CT, Collantes-Estevez E, Dougados M (2009). The development of Assessment of SpondyloArthritis international Society classification criteria for axial spondyloarthritis (part II): validation and final selection. Ann Rheum Dis.

[4] van der Linden S, Valkenburg HA, Cats A (1984). Evaluation of diagnostic criteria for ankylosing spondylitis. A proposal for modification of the New York criteria. Arthritis Rheum.

[5] Rudwaleit M, van der Heijde D, Landewe R, Akkoc N, Brandt J, Chou CT, Dougados M, Huang F, Gu J, Kirazli Y (2011). The Assessment of SpondyloArthritis International Society classification criteria for peripheral spondyloarthritis and for spondyloarthritis in general. Ann Rheum Dis.

[6] Gouveia EB, Elmann D, Morales MS (2012). Ankylosing spondylitis and uveitis: overview. Rev Bras Reumatol.

[7] Rosenbaum JT (2015). Uveitis in spondyloarthritis including psoriatic arthritis, ankylosing spondylitis, and inflammatory bowel disease. Clin Rheumatol.

[8] Robinson PC, Claushuis TA, Cortes A, Martin TM, Evans DM, Leo P, Mukhopadhyay P, Bradbury LA, Cremin K, Harris J (2015). Genetic dissection of acute anterior uveitis reveals similarities and differences in associations observed with ankylosing spondylitis. Arthritis Rheumatol.

[9] Martin TM, Zhang G, Luo J, Jin L, Doyle TM, Rajska BM, Coffman JE, Smith JR, Becker MD, Mackensen F (2005). A locus on chromosome 9p predisposes to a specific disease manifestation, acute anterior uveitis, in ankylosing spondylitis, a genetically complex, multisystem, inflammatory disease. Arthritis Rheum.

[10] O'Rourke M, Haroon M, Alfarasy S, Ramasamy P, FitzGerald O, Murphy CC (2017). The Effect of Anterior Uveitis and Previously Undiagnosed Spondyloarthritis: Results from the DUET Cohort. J Rheumatol.

[11] Lu MC, Hsu BB, Koo M, Lai NS (2017). Higher risk of incident ankylosing spondylitis in patients with uveitis: a secondary cohort analysis of a nationwide, population-based health claims database. Scand J Rheumatol.

[12] Yen JC, Hsu CA, Hsiao SH, Hsu MH (2017). Acute Anterior Uveitis as a Risk Factor of Ankylosing Spondylitis-A National Population-Based Study. Int J Environ Res Public Health.

[13] Oh BL, Lee JS, Lee EY, Lee HY, Yu HG (2018 ). Recurrent anterior uveitis and subsequent incidence of ankylosing spondylitis: a nationwide cohort study from 2002 to 2013. Arthritis Res Ther.

[14] Yang P, Wan  W, Du L, Zhou  Q, Qi J, Liang  L, Wang C, Wu  L, Kijlstra  A (2018 ). Clinical features of HLA-B27-positive acute anterior uveitis with or without ankylosing spondylitis in a Chinese cohort. Br J Ophthalmol.

[15] Juanola X, Loza Santamaria E, Cordero-Coma M, Group SW (2016 ). Description and Prevalence of Spondyloarthritis in Patients with Anterior Uveitis: The SENTINEL Interdisciplinary Collaborative Project. Ophthalmology.

[16] Jabs DA, Nussenblatt RB, Rosenbaum JT (2005 ). Standardization of Uveitis Nomenclature Working G: Standardization of uveitis nomenclature for reporting clinical data. Results of the First International Workshop. Am J Ophthalmol.

[17] Mitulescu TC, Popescu C, Naie A, Predeteanu D, Popescu V, Alexandrescu C, Voinea LM (2015 ). Acute anterior uveitis and other extra-articular manifestations of spondyloarthritis. J Med Life.

[18] Chung YM, Liao HT, Lin KC, Lin YC, Chou CT, Chen CH, Tsai CP (2009 ). Prevalence of spondyloarthritis in 504 Chinese patients with HLA-B27-associated acute anterior uveitis. Scand J Rheumatol.

[19] Rosenbaum JT, Rosenzweig HL (2012 ). Spondyloarthritis: the eyes have it: uveitis in patients with spondyloarthritis. Nat Rev Rheumatol.

[20] Thottam GE, Krasnokutsky S, Pillinger MH (2017 ). Gout and Metabolic Syndrome: a Tangled Web. Curr Rheumatol Rep.

[21] Haroon M, O'Rourke M, Ramasamy P, Murphy CC, FitzGerald O (2015 ). A novel evidence-based detection of undiagnosed spondyloarthritis in patients presenting with acute anterior uveitis: the DUET (Dublin Uveitis Evaluation Tool). Ann Rheum Dis.

[22] Khan MA, Haroon M, Rosenbaum JT (2015 ). Acute Anterior Uveitis and Spondyloarthritis: More Than Meets the Eye. Curr Rheumatol Rep.

[23] Rudwaleit M, Metter A, Listing J, Sieper J, Braun J (2006 ). Inflammatory back pain in ankylosing spondylitis: a reassessment of the clinical history for application as classification and diagnostic criteria. Arthritis Rheum.

[24] Sieper J, van der Heijde D, Landewe R, Brandt J, Burgos-Vagas R, Collantes-Estevez E, Dijkmans B, Dougados M, Khan MA, Leirisalo-Repo M (2009 ). New criteria for inflammatory back pain in patients with chronic back pain: a real patient exercise by experts from the Assessment of SpondyloArthritis international Society (ASAS). Ann Rheum Dis.

[25] Wang R, Crowson CS, Wright K, Ward MM (2018 ). Clinical evolution of patients with new-onset inflammatory back pain: a population-based cohort study. Arthritis Rheumatol.

[26] Wach J, Maucort-Boulch D, Kodjikian L, Iwaz J, Broussolle C, Seve P (2015 ). Acute anterior uveitis and undiagnosed spondyloarthritis: usefulness of Berlin criteria. Graefes Arch Clin Exp Ophthalmol.

[27] Cantini F, Nannini C, Cassara E, Kaloudi O, Niccoli L (2015 ). Uveitis in Spondyloarthritis: An Overview. J Rheumatol Suppl.

[28] Garcia-Vicuna R, Zarco P, Gonzalez CM, Vanaclocha F, Marin-Jimenez I, Cea-Calvo L (2016 ). Two-year incidence of psoriasis, uveitis and inflammatory bowel disease in patients with spondyloarthritis: A study in the AQUILES cohort. Reumatol Clin.

